# Dental Protective Association

**Published:** 1900-09-15

**Authors:** N. S. Hoff, Geo. L. Field


### DENTAL PROTECTIVE ASSOCIATION.

In view of the fact that misleading statements in regard to the attitude of the Dental Protective Association, in its relations to the International Tooth Crown Co., in its efforts to protect the dental profession from the unwarranted extortion of said International Tooth Crown Co , are being continuously circulated, even to the extent of a systematic and concerted effort by certain parties who find it to their pecuniary interest to discredit the work of Dr. J. N. Crouse as the chief executive of the Dental Protective Association; unjustly, as we believe, accusing Dr. Crouse of using the Dental Protective Association, its funds and membership, to further his own private business enterprises, we, the members of the Michigan Dental Association, in convention assembled, this 12th day of June, 1900, at Kalamazoo, adopt the following resolution expressive of our confidence in the work of Dr. Crouse in our behalf through the Dental Protective Association:

*Resolved*, That we, as the legally organized association of the members of the dental profession of the State of Michigan, do hereby express to the public in general, and to Dr. J. N. Crouse in particular, our cordial approval of his conduct of the business management of the affairs of the Dental Protective Association, approving of his past conduct of the affairs of the Association, of which a majority of us are members. We believe he has honestly and conscientiously regarded and placed the interests of the profession above all personal pecuniary or ambitious ends, and we do freely commit the conduct of our affairs to his management for the future, pledging our material and moral support in every possible way.

Signed, { N. S. Hoff,<br>Geo. L. Field.

---

Dr. J. C. Schuller, of Center, Texas, says that very good saws for the engine can be obtained by applying to a jeweler, and asking him to attach ratchets from old watches to the end of engine points. He says they are of the highest tempered steel with saw edges and well sharpened, of various sizes and thicknesses, and that the jeweler can afford to furnish them, mounted, for about ten cents each.—*Items of Interest for July.*

